# Ectopic Craniopharyngioma Recurrence: A Case Report and Literature Review

**DOI:** 10.7759/cureus.69607

**Published:** 2024-09-17

**Authors:** João Nogueira, Joana Sobreiro Silva, Renata Marques, Cristiano Antunes, Renato Pereira, Miguel Afonso Filipe

**Affiliations:** 1 Neurosurgery, Hospital de Braga, Braga, PRT; 2 Anatomic Pathology, Hospital de Braga, Braga, PRT; 3 Neurosurgery, Centro Hospitalar Tondela-Viseu, Viseu, PRT

**Keywords:** adamantinomatous craniopharyngioma, craniopharyngioma, ectopic craniopharyngioma, ectopic recurrence, neoplasm metastasis, neoplasm seeding

## Abstract

Craniopharyngiomas are tumors of the central nervous system, typically located in the sellar/parasellar region. Despite being benign, they behave aggressively due to their propensity to invade nearby important structures, making total resection challenging. Distant spread of craniopharyngioma is a rare but significant complication. Most cases result from spread along the surgical path, while others result from dissemination along the cerebrospinal fluid (CSF) pathways.

We report a case of a parasellar adamantinomatous craniopharyngioma with progressive visual loss. The patient was operated on through a right pterional craniotomy three times due to recurrence. After the last surgery, fractionated stereotactic radiotherapy was performed on the tumor residue. On follow-up brain MRI, a new extra-axial lesion was found in the left frontal region with solid and cystic components, with apparent dural implantation. Left frontal craniotomy was performed, and the lesion was excised with resection of its dural implant. Histological findings revealed it to be adamantinomatous craniopharyngioma, grade 1, according to the World Health Organization (WHO).

Despite being rare, craniopharyngioma ectopic recurrence is a possible surgical complication. Despite the poorly understood mechanism, the literature highlights the importance of paying attention to tumor spillage during surgery to prevent distant recurrences.

## Introduction

Craniopharyngiomas are benign (World Health Organization (WHO) grade 1) epithelial tumors arising from embryologic squamous remnants of the craniopharyngeal duct or Rathke's pouch. They represent 0.8% of all brain tumors, with an incidence of 0.19 per 100,000 [1]. They have a bimodal age distribution, with a childhood peak age between five and 15 and an adult peak age between 45 and 60 [2].

There are two histologic types: adamantinomatous and papillary. Although both tumors arise in the same region and display squamous lineage, recent consensus is that they are distinct tumors with differing epidemiology, radiology, histology, and molecular genetics [3]. Adamantinomatous craniopharyngiomas are usually lobulated and cystic sellar and suprasellar lesions. Calcification, ossification, fibrosis, and cholesterol deposits are frequently present. On the other hand, papillary craniopharyngiomas are less often cystic and rarely have calcifications [4]. They often demonstrate local brain invasion and may adhere to adjacent vessels and nerves, making it extremely hard to achieve a total resection with recurrence rates as high as 57% [5].

Common presentations include visual disturbances and endocrine dysfunction. Cognitive impairment, personality change, and symptoms of elevated intracranial pressure have also been described.

The treatment of craniopharyngiomas typically involves surgical resection, sometimes accompanied by radiation therapy. There is ongoing debate about whether to opt for complete surgical resection alone or to combine a more limited resection with radiation therapy. While local recurrence is frequently seen after surgical resection, ectopic recurrence is rare and scarcely reported in the literature. Two mechanisms of spread have been described: seeding along the surgical tract or through the cerebrospinal fluid (CSF) [6].

In this article, we report on a 77-year-old male with ectopic recurrent craniopharyngioma and a review of the literature.

## Case presentation

**Figure 1:**
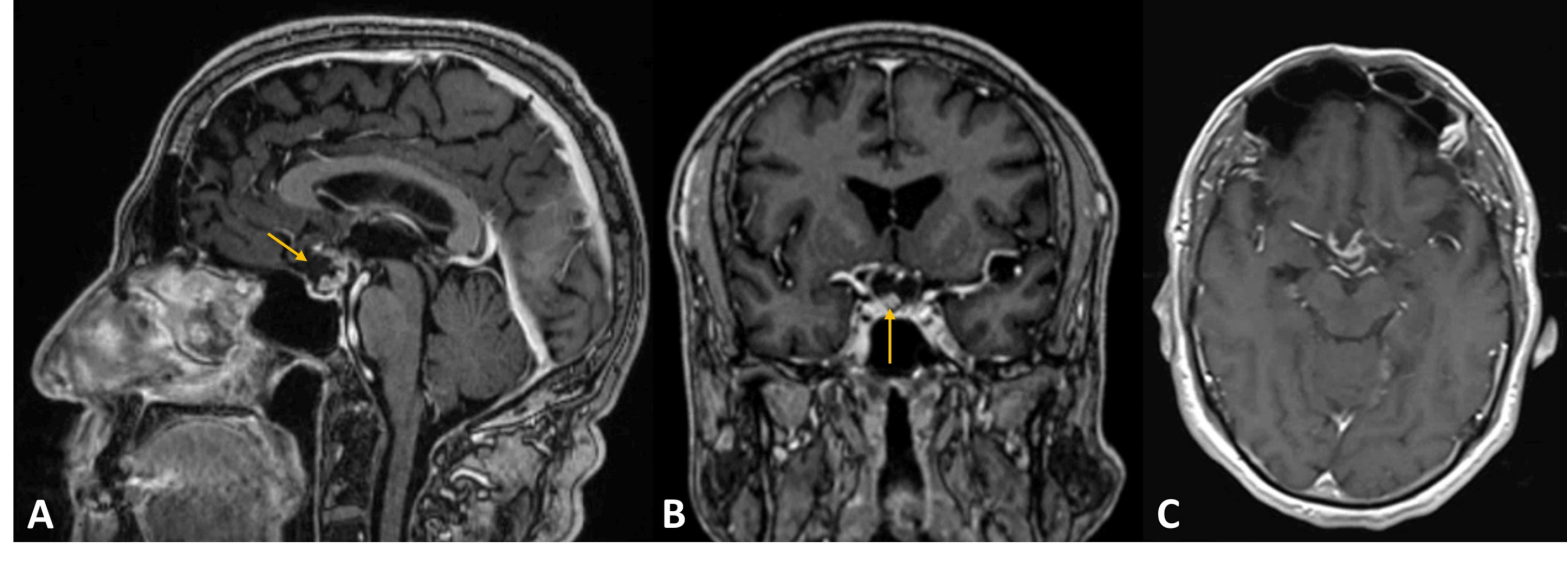
Preoperative MRI Sellar/parasellar extra-axial lesion with solid and cystic components. Contrast enhancement of the solid element and cystic wall. Gadolinium-enhanced T1-weighted images: (A) sagittal, (B) coronal, and (C) axial. Yellow arrows indicating the lesion.

A 77-year-old male with complaints of progressive visual loss, diagnosed with bitemporal hemianopsia, was studied with a brain MRI. A sellar and suprasellar extra-axial lesion with 15.5×12 mm was identified, compressing the optic chiasma. It had mixed solid and cystic components. The solid component and the cystic wall enhanced heterogeneous with contrast (Figure 1).

The patient underwent a right pterional approach and a gross total lesion resection. His vision improved after surgery. The histologic examination revealed epithelial tissue with sizeable cystic component, squamous epithelial trabeculae, basaloid cells, stellate reticulum, and nodules of wet keratin compatible with adamantinomatous craniopharyngioma, grade 1 (WHO 2021).

Three years later, and with a new bilateral visual deficit with an almost amaurotic right eye, he was investigated with a new MRI, which revealed a significant tumor recurrence. The pterional craniotomy was performed again, and the lesion was partially resected, decompressing the optic chiasm and the optic nerves. Despite that, the vision in his right eye did not improve.

One year later, he started to lose vision in his left eye. A new tumoral recurrence was diagnosed, and he underwent a third surgery through the previous right pterional craniotomy, resulting in new partial resection and decompression of the left optic nerve and optic chiasma, sparing the remaining vision of his left eye. After, he underwent radiotherapy for the suprasellar residual lesion.

**Figure 2:**
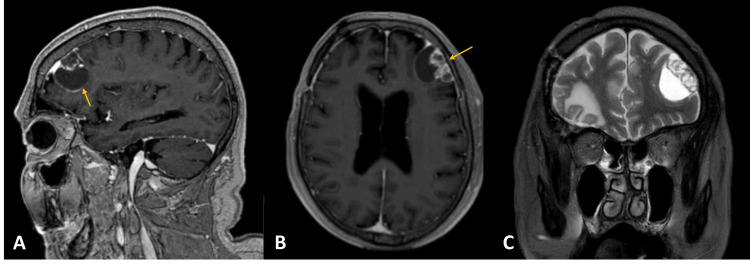
Cranial MRI showing ectopic recurrence Left frontal lobe extra-axial lesion with a solid and cystic component, with heterogeneous contrast enhancement. (A) Gadolinium-enhanced sagittal T1-weighted image; (B) gadolinium-enhanced axial T1-weighted image; (C) T2-weighted coronal image. Yellow arrow indicating the lesion.

However, eight months after the last surgery, a routine brain MRI demonstrated a new multicystic left frontal lesion, apparently extra-axial (Figure 2).

**Figure 3:**
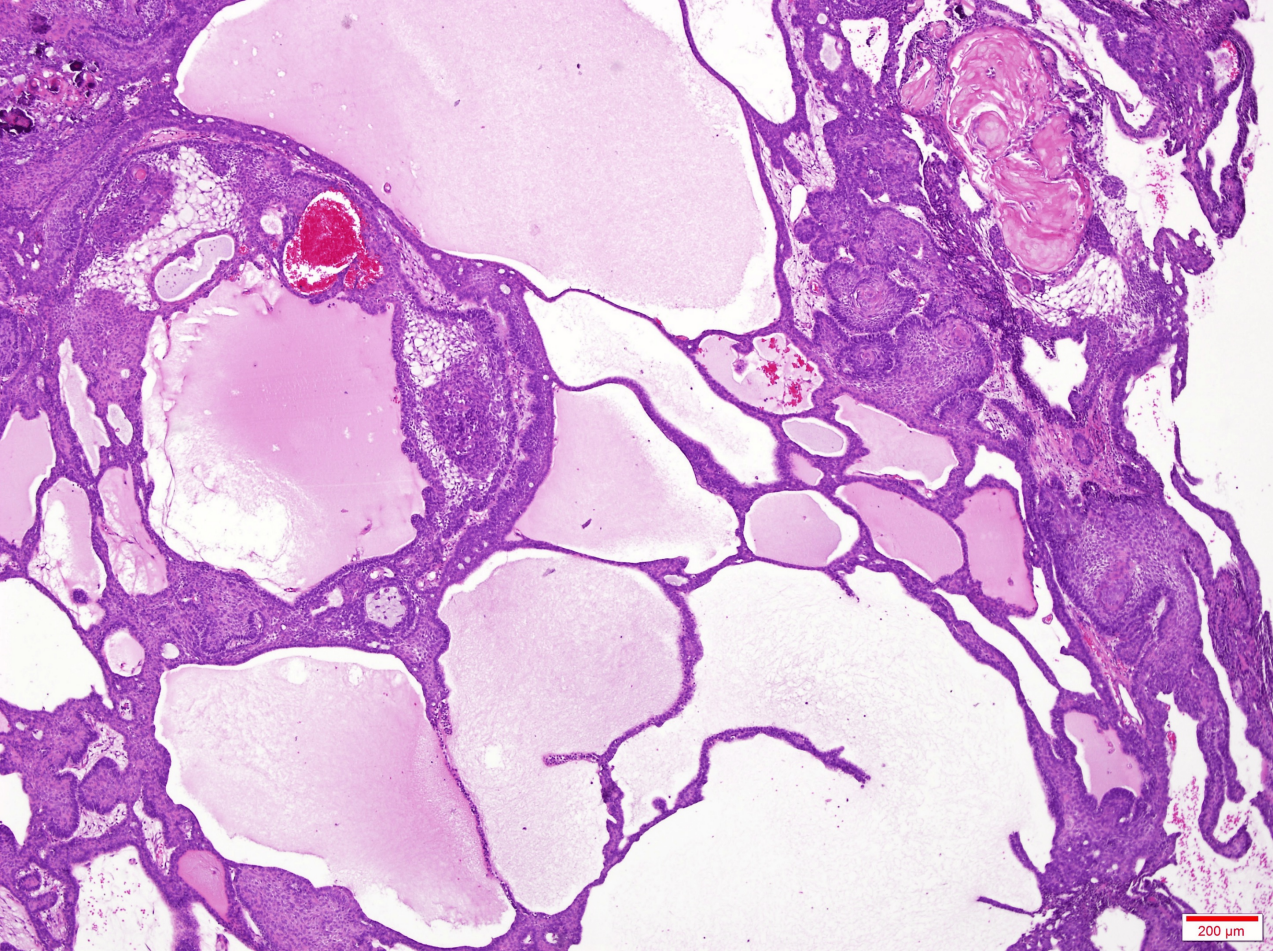
Histopathological results showing adamantinomatous craniopharyngioma H&E at 40×. Mixture of solid and cystic areas, with the cysts filled with a proteinaceous material that stains pink with H&E. H&E: hematoxylin and eosin

A left frontal craniotomy was made, and the extra-axial lesion was fully resected. Intraoperatively, the dura was involved by the lesion, which was also excised. The pathological staining confirmed adamantinomatous craniopharyngioma (Figure 3).

The patient had no new deficits following surgery. A cerebral MRI conducted six months post-surgery showed no evidence of recurrence of the metastatic lesion and stability of the sellar craniopharyngioma.

## Discussion

Ectopic or metastatic recurrent craniopharyngioma is extremely rare. Up to date, only 66 patients with 68 cases have been described, including ours (Table 1) [5-55].

**Table 1:** Review of cases of ectopic recurrent craniopharyngioma NM: not mentioned; STR: subtotal resection; GTR: gross total resection; PR: partial resection, gamma knife; M: male; F: female; RT: radiotherapy; CPA: cerebellopontine angle

Author, year	Age (years), gender	Histology	Previous treatments	Ectopic recurrence location	Ectopic recurrence mechanism	Interval between the first surgery and ectopic recurrence (years)
Present	77, M	ACP	Cranio GTR (first); cranio STR (second); cranio STR+RT (third)	Left frontal lobe	CSF	4
Carfagno et al. (2023) [7]	17, M	NM	Cranio GTR	Right ventricle	CSF	10
Steed et al. (2023) [8]	10, F	ACP	Cranio STR+RT (first); cranio STR (second)	Dorsal (T1)	CSF	3.5
Selfa et al. (2023) [9]	7, M	ACP	Cranio STR (first); cranio STR+RT (second)	CPA	CSF	20
	36, M	ACP	Cranio GTR	Cerebellum	CSF	18
Ji et al. (2023) [10]	63, F	ACP	Cranio STR (first); TSS STR (second)	Right temporal lobe	CSF	3
	49, F	ACP	Right	Right frontal lobe	Surgical approach	7 (first); 16 (second)
Cai et al. (2019) [5]	28, M	ACP	Cranio GTR	Right temporal lobe	CSF	1
Renfrow et al. (2018) [11]	14, F	ACP	Cranio GTR (first); cranio GTR (second); RT (third)	Left lateral ventricle	CSF	12
Mahdi et al. (2018) [12]	24, M	ACP	Cranio GTR	Right CPA	CSF	1
Jian et al. (2017) [13]	42, M	PCP	Cranio GTR	Right frontal lobe	CSF	0.3
Carleton-Bland et al. (2017) [14]	10, M	ACP	Cranio STR (first); cranio STR+RT (second)	Right lateral ventricle	CSF	2
Du et al. (2016) [15]	6, F	ACP	Cranio GTR	Right frontal lobe	Surgical approach	5
	4, M	ACP	Cranio GTR	Fourth ventricle	CSF	4
Clark et al. (2015) [16]	33, F	ACP	Cranio GTR (first); cranio GTR (second)	Left Sylvian fissure	Surgical approach	34
Yang et al. (2015) [17]	35, M	NM	Cranio GTR	Right frontal lobe	Surgical approach	9
	46, M	PCP	Cranio GTR	Interhemispheric	Surgical approach	2
	42, M	ACP	Cranio GTR (first); cranio GTR (second)	Right frontal lobe	Surgical approach	6
Gonçalves et al. (2014) [18]	49, M	ACP	Cranio GTR	Right frontal lobe	Surgical approach	5
Jakobs and Orakcioglu (2012) [19]	61, F	ACP	Cranio GTR (first); RT (second)	Right frontal bone	Surgical approach	11
Elfving et al. (2011) [20]	4, F	ACP	Cranio GTR (first); cranio STR+RT (second)	Right frontal lobe	Surgical approach	11
Salunke et al. (2011) [21]	5, F	NM	Cranio STR	Right Sylvian fissure	Surgical approach	0.92
de Blank and Minturn (2011) [22]	5, F	NM	Cranio GTR (first); RT (second)	Left CPA	Surgical approach	17
Kordes et al. (2011) [23]	7, M	ACP	Cranio PR+TSS+RT	Right parietal lobe	Surgical approach	1.25
Wang et al. (2010) [24]	3, M	ACP	Cranio GTR+RT	Right frontal lobe	Surgical approach	2
Lermen et al. (2010) [25]	45, M	ACP	Cranio STR (first); cranio GTR (second)	Lumbar space	CSF	0.5
Schmalisch et al. (2010) [26]	11, M	ACP	Cranio GTR	Right Sylvian fissure	Surgical approach	2
	23, F	ACP	Cranio STR (first); TSS (second)	Right frontal lobe	Surgical approach	4
	32, M	ACP	Cranio GTR	Right parietal lobe	CSF	10
Romani et al. (2010) [27]	18, F	ACP	Cranio GTR	Interhemispheric	Surgical approach	4
Frangou et al. (2009) [6]	10, M	ACP	Cranio STR (first); cranio STR (second); cranio STR+RT (third)	Right parietal lobe	CSF	4
Elliott et al. (2009) [28]	3, F	ACP	Cranio GTR	Prepontine cistern	CSF	10
	2, M	ACP	Cranio STR (first); cranio GTR (second); cranio GTR (third)	Left CPA	CSF	3.5
	3, F	ACP	Cranio GTR	Right Sylvian fissure	Surgical approach	1.67
	6, M	ACP	Cranio STR (first); cranio GTR (second)	Left frontal lobe	CSF	7.08
Bikmaz et al. (2009) [29]	37, F	NM	NM	Prepontine	CSF	15
	32, M	NM	GTR	Right frontal lobe	Surgical approach	9
	12, M	NM	STR+RT	CPA	CSF	12
Novák et al. (2008) [30]	48, M	NM	NM	Posterior fossa	CSF	19
Jeong et al. (2006) [31]	8, F	ACP	Cranio GTR	Right frontal lobe	Surgical approach	4
Yamada et al. (2006) [32]	17, F	ACP	Cranio STR+RT	Left frontal lobe	CSF	5
Bianco et al. (2006) [33]	27, F	ACP	Cranio STR (first); cranio GTR (second)	Left temporal lobe cortex	Surgical approach	10
Kawaguchi et al. (2005) [34]	50, F	NM	NM (first); cranio GTR (second)	Left frontal lobe	Surgical approach	2
Ishii et al. (2004) [35]	2, M	NM	Cranio STR (first); cranio GTR (second)	Right frontal lobe	Surgical approach	0.17
Liu et al. (2002) [36]	65, F	NM	Cranio GTR	Right frontal lobe	Surgical approach	3
Nomura et al. (2002) [37]	17, F	ACP	Cranio STR (first); cranio STR+RT (second)	Right frontal and temporal lobe	CSF	3.92
Fuentes et al. (2002) [38]	32, M	ACP	GTR	Right frontal lobe	Surgical approach	5
	11, M	ACP	GTR	NM	Surgical approach	3
	9, M	ACP	GTR	Right frontal lobe; right temporal lobe	Surgical approach	10
Elmaci et al. (2002) [39]	62, F	PCP	Cranio GTR	Left temporal lobe	CSF	2
Novegno et al. (2002) [40]	6, M	ACP	Cranio GTR	First left frontal lobe; second left pontine R cerebral basal	1st surgical approach; 2nd CSF	3 (first); 4 (second)
Lee et al. (2001) [41]	26, M	PCP	Cranio STR+GK	Lumbar space	CSF	1.58
Ito et al. (2001) [42]	62, M	ACP	Cranio GTR	Right frontal lobe	CSF	3
Freitag et al. (2001) [43]	61, F	NM	NM	Right frontal lobe	Surgical approach	5
Kim et al. (2001) [44]	Child, NM	NM	NM	Internal auditory canal	CSF	NM
Gupta et al. (1999) [45]	73, M	ACP	Cranio GTR	Left parietal lobe and left frontal lobe	CSF	7
Lee et al. (1999) [46]	31, M	NM	Cranio GTR	Right frontal lobe	Surgical approach	5
Israel and Pomeranz (1995) [47]	12, M	NM	Cranio GTR	Right frontal lobe	Surgical approach	2
Keohane et al. (1994) [48]	7, F	NM	Cranio STR+RT	Left CPA	CSF	26
Tomita and McLone (1993) [49]	Child, NM	NM	NM	Right frontal lobe	Surgical approach	NM
Malik et al. (1992) [50]	6, M	ACP	Cranio PR+RT (first); 2nd cranio PR (second); cranio GTR (third)	Right frontal lobe	Surgical approach	21
Tomita et al. (1992) [51]	23, F	NM	Cranio PR (first); RT (second)	Right CPA, interpeduncle, prepontine	CSF	25
Gökalp et al. (1991) [52]	3, M	NM	Cranio GTR	Fourth ventricle	CSF	20
Ragoowansi and Piepgras (1991) [53]	47, M	NM	Stereotactic biopsy (first); cranio GTR (second)	Right Sylvian fissure	Surgical approach	1
Barloon et al. (1988) [54]	5, M	NM	Cranio STR RT (first); cyst aspiration+RT	Right frontal lobe	Surgical approach	5
Baba et al. (1978) [55]	7, F	NM	Cranio STR	Prepontine C3	CSF	7

The literature describes two mechanisms of spread: seeding along the surgical tract, which is attributed to iatrogenic contamination of brain tissue with tumor cells during the process of removing parts of the tumor, and dissemination via CSF with metastasis that have no relationship with the surgical tract [5,6,25].

The majority of cases (35) were related to the surgical tract, and 33 were due to seeding through the CSF pathway. The most common site of ectopic recurrence is, by far, the frontal lobe (29 cases). This may be due to transcranial surgery for craniopharyngioma via the pterional approach being the most frequently used, with local cell dissemination during operation.

Despite our case being also a frontal ectopic craniopharyngioma, the proposed dissemination mechanism was most probably due to dissemination by CSF because it is on the contralateral side of the approach. Other locations (temporal, parietal cerebellopontine angle, etc.) were also associated with CSF dissemination. The lumbar spine was the most faraway location reported [10,15]. Most of these lesions were extra-axial. These suggest that the meninges may serve as a base for the implantation of craniopharyngioma cells. Also, as demonstrated in some cases, marked bony erosion may be seen [6].

Craniopharyngiomas have a bimodal age distribution. Thirty-four cases were found in children and 32 cases in adults. This distribution suggests that ectopic recurrences don't seem to be confined to a particular age group. The mean age in children was 7.44 (±4.14) and in adults 43.69 (±14.54). Our case, at 77 years old (81 at ectopic diagnosis), is the oldest reported. Male gender was more frequent, with 37 cases reported vs. 26 on women; in other cases, gender was not reported. This suggests potential gender-related factors in the occurrence of ectopic craniopharyngioma.

Only four metastatic craniopharyngiomas were papillary, which is no surprise since it is the less frequent type. Also, the papillary type usually has a more indolent course with lower recurrence rates after total resection [14]. In 21 cases, the pathology was unclear, especially in older reports.

Gross total resection does not seem to prevent metastatic disease. It may even be associated with more recurrence since more aggressive surgery and longer operative time allow more tumor tissue to be manipulated and exposed to either the surgical tract or CSF pathways [16]. This highlights the need for protection of the surgical field, cyst aspiration before lesional excision, careful manipulation of the tumor during surgery, and extensive irrigation with saline throughout the surgery to prevent contamination with tumor cells [33,42].

Postoperative radiotherapy remains controversial as a treatment option for craniopharyngiomas [3]. It is usually indicated in cases where total gross resection is not possible. As in this case report, another 18 patients received postoperative adjuvant radiotherapy and still had ectopic recurrence. It seems that radiotherapy is not protective against ectopic recurrence.

## Conclusions

The treatment and recurrence of craniopharyngiomas present significant challenges. While surgical resection, sometimes combined with radiation therapy, remains the primary treatment approach, there is ongoing debate about the best strategy to minimize recurrence.

Local recurrence is common, but ectopic recurrence is rare and can occur through seeding along the surgical tract or dissemination via CSF. The frontal lobe is the most frequent site of ectopic recurrence, likely due to the common use of the pterional approach in surgery.

Ectopic recurrences are not confined to a specific age group, though they appear more frequently in males. 

Despite aggressive surgical techniques and the use of postoperative radiotherapy, ectopic recurrences still occur, highlighting the need for meticulous surgical practices and further research into effective preventive measures.
